# Pathology and Treatment of Exposed Pulps

**Published:** 1875-11

**Authors:** W. B. Knapp


### DENTAL REGISTER.

VOL. XXIX.] NOVEMBER, 1875. [NO. 11.

### PATHOLOGY AND TREATMENT OF EXPOSED PULPS.

BY DR. W. B. KNAPP.

*Read before the Indiana State Dental Society.*

Previous to entering upon the pathology and treatment of exposed pulps, let us first consider the histology of the teeth and their physiological aspect briefly.

The teeth are composed of three hard elementary tissues, enclosing a soft fleshy mass in the center; the enamel, dentine, cementum and pulp.

The enamel forms the covering of all the free portion of the tooth, it is the hardest tissue in the body, containing in the adult about 95 per cent of earthy matter. In structure it consists of a series of hexagonal prisms or fibres running with a somewhat wavy course, but generally at right angles with the periphery of the dentine of the crown, upon which they rest with one extremity, the other being free.

The enamel seems to be destitute of nutrient vessels and nerves; and Salter says: "There is every reason to believe that the enamel is wholly destitute of vitality, that it undergoes no nutrient changes after it is once formed, and that it is only influenced by the physical and mechanical changes to which it is exposed."

There are some facts that lead me to doubt the correctness of this theory. If it is true, why should the enamel of an adult contain more earthy matter than that of a child? Or, as all physical and mechanical agencies must be external, why should the enamel of a devitalized tooth deteriorate so much more rapidly than that of a live one?

Tomes while saying nothing in regard to the vitality of the enamel, does say: "Although the fibrils have attained their full length some time before the tooth is cut, the development of the tissue can scarcely be regarded as matured until after that period; for at the time a tooth passes through the gum, the enamel is comparatively soft and fragile, and it is only after the lapse of some months or even years, that it attains its full degree of hardness."

The enamel is thickest upon the masticating surface of the tooth, it terminates at the neck, where it is overlapped by the cementum.

The dentine or tooth ivory, which constitutes the bulk of the tooth, is a tissue of bony hardness, about 75 per cent of its weight consists of earthy matter. The minute histology of the dentine has not been fully demonstrated, and the diversity of opinion among authorities is such, that if one is right others must be entirely in error, and vice versa. But in general terms, the dentine consists of a series of minute tubes radiating from a central cavity containing the pulp, between the tubes is a hyaline homogeneous mass, called the intertubular tissue. The tubes pursue a wavy or spiral course, are dichotomous, the branches anastomose freely and terminate at the periphery of the dentine in loops or fine net work. These tubes do not, it is generally believed, pass into the enamel. But Tomes says: "In the crown of the teeth, they terminate by forming loops, or become too minute to be traced, or pass into the enamel and are lost." Again: "By the extension of the dentinal tubes into the enamel, and into the cementum, a connection is formed more intimate than the mere superposition and adhesion of the one to the other would have established, and the more so as the three tissues are developed from three different formative pulps." In the roots however it is different, as they frequently pass into the cementum, particularly near the apex, uniting with the lacunæ of the cementum.

Salter produces a very fine argument to prove that these tubes are filled with a dense fluid or plasma. In this he is supported by Koelliker and others. But I prefer and shall adhere in my paper to the theory of Tomes, namely, "That each dentinal tube is tenanted permanently by a soft fibril, which, after passing from the pulp into the tube, follows its ramifications," but, whether plasma or fibril, all agree that it is the office of the tubes to convey nutrition from the pulp to the hard tissues.

Primary dentine is nonvascular except as an abnormal condition, but secondary dentine is highly vascular. "Dentine exhibits vital phenomena which show it to be a part of the living body. First, it is sentient; second, it is susceptible of nutritional changes; third, it takes cognisance, so to speak, of injuries done to its outer surface, leading to a renewal of ivory growth on the pulp surface."

Cementum is a structure closely resembling true bone. It covers over the roots of the teeth as the enamel does the crown. It contains 70 per cent of earthy matter, and is generally non-vascular; cementum presents the vital phenomena even more markedly than the dentine. It is formed from the periosteum after the manner of true bone, and from it receives the most of its nutrition and nervous influence, also receiving some from the pulp through the dental tubuli which to a limited extent penetrate it. The periosteum is a fibro vascular, non-elastic connective tissue, lining the socket of the tooth and surrounding alveolar process. "The pulp is a soft mass, which exactly fills the chamber in the crown and fangs of the tooth, it is of a pinkish color and is rather translucent. It contains a large amount of fluid and dries to a mere film, nerves and blood vessels abound throughout its structure."

The teeth are generally regarded as a cutaneous production, being developed from sub-mucous papillæ, enveloped or overlaid by mucous or epithelial membrane. The papillæ consist of a homogeneous, subgranular blasma or matrix, in which vessels and nerves are gradually developed, forming the dentinal pulp. The epithelial cells of the overlaying membrane become columnar in form, assuming the position ascribed to the enamel prisms and form the matrix of that tissue. This matrix seems to be destitute of vessels and nerves, and I agree with Garretson in thinking that calcification is carried on by an endosmosis from the dentinal pulp, of earthy matter, which is deposited in these cells. This view, although not the generally received one, explains some changes which take place in the enamel of teeth, that are fully erupted, that I think can be explained in no other way.

In studying the development of the pulp, it is found that upon the formative pulp, there is arranged a series of columnar cells, constituting the membrana-eboris from the distal extremities of which minute tubular threads project, and as the dentine forms from without inwards, these are prolonged centripetally, a homogeneous blastema separating them, and being calcified with them. These prolongations are the dentinal tubes and the blastema is the inter-tubular tissue. These tubes as I have previously stated, I believe to be tenanted by soft fibrils. Thus it will be seen that the enamel, dentine and cementum, are built up by a formative action of the pulp and soft tissues. This is a normal function of these parts, commencing with the first centers of ossification and continuing actively, until the full development of the tooth has been reached. Here it ceases with the soft tissues, excepting that the cementum receives its nourishment in part from its surrounding membrane. Not so however with the pulp, which continues throughout its life, to not only afford nutriment to the tooth, and remove the debris, the result of the change continually taking place in all organized tissue, but also continues its formative function, gradually changing the constituents of the tooth, adding mineral salts until the teeth become very much denser and less subject to those influences that promote decay.

So well understood is this, that the argument is frequently used with young people, that if their teeth can be saved a limited period, they will probably have little trouble afterwards.

Also slowly diminishing the diameters of the pulp cavities and the canals of the roots, this continues to such an extent, that under purely normal conditions, in old people, these will frequently be found to be nearly obliterated. But exciting causes often stimulate the pulp to greater than merely normal activity. Where abrasion of a tooth is so great as to cause the nerve to be unfavorably affected by external agents as pressure or thermal changes, the pulp rapidly retires before the thickening walls with which it lines its chamber, as an additional barrier and protection, and the tubuli over the weak point are closed by calcific deposit, forming a structure harder than ordinary dentine.

If the enamel be removed by the file or disc, from a portion of a tooth, and that portion be kept perfectly cleaned and polished, the dentine soon presents a semi-transparency, and becomes much harder in its texture, the tubuli have been obliterated to a considerable depth by calcific deposit, due to the formative action of the pulp, through the fibrilla. So well is this understood, that some dentists prefer to remove superficial decay and shallow cavities with the file, disc or chisel, to excavating and filling. Some even advocate the permanent separation of the teeth by these means, to prevent the retention of destructive agents at the points of contact, insisting that exposed dentine will become by calcific deposit, so hard and dense, that if kept clean, it will resist decay better than enamel, where in contact upon the proximate surfaces of the teeth.

In slow decay, in cases of arrested decay; and in cavities that have been filled with metals, we often find a belt or zone of considerable thickness, where the tubuli have become obliterated and the dentine is much denser than normal. In excavating cavities for the purpose of filling, we often find underneath the debris of decay, a new layer of dentine, distinctly separated from the overlaying decayed dentine, by well marked lines of separation, there being frequently an apparent union between the two over considerable space. In these cases the decay had undoubtedly reached the pulp, but it being protected from the influence of external changes, by the overlaying mass of decayed matter, has secreted lime salts, in sufficient quantities, to build a new wall of healthy tissue, for its protection from the approaching enemy. How often or under what conditions this may occur, I have no means of knowing, but a sufficient number of cases have been observed and noted by careful and reliable operators, to establish the fact and I think, I have given the correct explanation of them.

Thus it will be seen that this delicate organ has considerable power of self-protection; and of raising new barriers, to resist the encroachment of decay, and where the decay has not advanced so rapidly as to expose it.

When the pulp becomes exposed, if left to itself, it soon becomes irritated and takes on destructive inflammation, leading to the death of the organ, and finally to an abscess. But a few years ago these were considered sufficient grounds to justify the extraction of the tooth. Now no one thinks of such a procedure.

Abscesses can be treated and healed, dead pulps removed, the pulp cavity purified and filled and the tooth saved for a considerable length of time, sufficient to justify the expense and labor. The length of time these dead teeth can be kept, depends upon many modifying conditions as the density and quality of the tooth structure, the nature of the secretion of the mouth and the amount of pressure they are required to resist in mastication.

But as a rule, necrosed teeth, especially the bicuspids and molars, soon become very friable and are easily broken down and lost. On the other hand, so long as the pulp is preserved, the tooth retains its normal condition and may last during life.

Now can not skill and science surround an exposed pulp with conditions so favorable, that its life will be preserved? Or after skill and science have exhausted their resources, will irritation continue, resulting in inflammation and ultimate death of the part?

It must be confessed that down to a late period all efforts to save exposed pulps, have resulted in pretty uniform failure. Nor is it difficult to understand the reasons why they failed.

The cap was usually a thin plate of some hard substance, such as lead, tin, gold, rubber or quill, and place it as carefully as might be over the exposure, such a cap could never be made to perfectly cover the orifice of exposure without leaving spaces between it and the surface of the pulp, it being contrary to physiological laws that there should be such spaces, the pulp would protrude into them, thus causing irritation and inflammation from its abnormal shape and position.

Sometimes the force used in filling would press the cap down upon the pulp, so producing the same result. Another fruitful source of trouble was the placing of metal fillings over metal caps, thus making good conductors of thermal changes and producing irritation and death from frequent shocks.

Other substances were tried as gold beater's skin, thin cork, papers, wool fibers, Hill's stopping and other preparations of gutta percha, etc., perhaps with occasional success, but those previously named were the materials generally resorted to, and they were all open to the same objection, namely, lack of adaptability. And the failures were so universal, that the practice was almost entirely discontinued.

Still I am not prepared to admit the hypothesis, that irritation must inevitably ensue, and the part die. In spite of failures the theory is still tenable, that if the pulp can be surrounded with conditions approximating those under which it is found normally, it will continue its normal functions.

The conditions essential for the preservation of the pulp are:

1st. The removal of all external sources of irritation, as decayed dentine and foreign substances in contact with the pulp.

2d. It is essential to subdue all inflammation of the pulp and dentine.

3d. The material used for capping should be plastic, of such a semi-fluid consistency as to mould itself perfectly, without pressure to the pnlp and dentine. It should harden promptly and sufficiently to withstand all necessary pressure in filling, it should unite firmly enongh to the walls of the cavity to retain its position. It should be a non-conductor and a non-irritant. We have no agent at present which will fulfill all of these conditions; that which approaches most nearly, is the oxy-chloride of zinc, which fills all but the last. The chloride of zinc is a powerful irritant.

It is to be hoped that ere long, chemistry will come to our aid, with some agent which will meet all the desired requirements.

At the present time many able practitioners decry the practice of capping pulps, maintaining that it is better policy to destroy them, and treat them as dead pulps. Claiming that they can not be successfully capped, and classing oxy-chloride of zinc with the other materials used.

This however is not doing justice to it, for it combines more of the requisite conditions than any other known substance, and has only one drawback, and in the majority of cases the pulp can be wholly or nearly protected from the irritant effects of the chlorine.

The advantages to be gained by the preservation of the pulp have been previously stated, and are so evident, and so great, that any means that gives fair prospect of success in favorable cases only, should be used at least in those favorable cases. A true physician always says "that while there is life, there is hope."

And in these cases if the operation fails to preserve the life of the pulp, the tooth is only in the same condition it would be in had the pulp been devitalized with arsenic, excepting the danger that might arise from the absorption of the arsenic.

It is stated upon the authority of some of our most intelligent and reliable operators, scattered over different parts of the country, that with cases of exposed pulp where there has been little or no inflammation, they are successful in nearly every case in preserving it.

In cases where there is a great deal of irritation, inflammation and even congestion, they are successful in the majority of instances in allaying the inflammation and reducing the pulp to its normal condition, and then usually have little trouble in capping it. Some even go further, and where suppuration has commenced, endeaver to, and claim that they are successful in sloughing off or excising the dead portion, restoring the rest to a healthy condition, and capping it.

I myself have seen some marked cases of this mode of treatment, but have not seen it carried to the extent practiced by some. Notably perhaps among whom Dr. J. Taft argues that if he can save a portion of the pulp in the root of a tooth he is accomplishing a desirable end, as he is preserving the life and nutrition of at least a part of the tooth. The question now arises, having fulfilled the conditions demanded by the theory, is that theory verified? To this, in the absence of better authority at my command, I will answer, that I have seen a number of cases in which I knew the pulp to have been fully exposed, some of them over considerable area, when capped, over which when the capping was removed, there was found to be a septum of secondary dentine, or amorphous mass of calcific deposit of varying thickness, similar to that previously spoken of.

When I say a number of cases, I mean six or eight, not a very large number, but enough to fully verify the theory. And having during the last five or six years capped or assisted in capping hundreds of pulps, very few of which have ever given evidence of irritation or death, the presumption is that they have protected themselves by calcific deposit, or at least maintained a normal condition under the cap.

There are many attendant circumstances, and pathological conditions that must not be overlooked, and that must modify the course of treatment of exposed pulps.

But time will not allow me to do more than name some of them at present. A very frequent cause of failure is the irritation kept up by the nutrient calcification of the pulp, for this there is no remedy: But there are many systemic conditions some of which can be palliated by systemic treatment that render it difficult to cap pulps Among these are the malarial poisons, scrofula, syphilitic taints, nervous prostration neuralgia, and the many unsatisfactory conditions of health arising from enfeebled nutrition.

### NUTRITION.

